# The health-related quality of life in children with IgA vasculitis

**DOI:** 10.3389/fped.2025.1534687

**Published:** 2025-05-14

**Authors:** Juan Zhou, Min Nie, Zixia Song, Xing Shen

**Affiliations:** ^1^ Department of Pediatrics, Bazhong Central Hospital, Bazhong, Sichuan, China; ^2^ Department of Pediatrics, Sichuan Clinical Research Center for Birth Defects, The Affiliated Hospital of Southwest Medical University, Luzhou, Sichuan, China; ^3^ Department of Pediatrics, NanChong Central Hospital, NanChong, Sichuan, China

**Keywords:** health-related quality of life, IgA vasculitis, children, PedsQL^TM^ 4.0, recurrence

## Abstract

**Introduction:**

The objective of this study was to investigate the health-related quality of life (HRQOL) in children diagnosed with IgAV and explore factors influencing it, aiming to provide insights for rehabilitation of these children.

**Methods:**

This study enrolled 114 children diagnosed with IgAV at Bazhong Central Hospital between January 2019 and January 2023. 51 healthy children undergoing health check-ups during the same period were enrolled in control group. Data on sociodemographic and clinical characteristics, as well as health-related quality of life scores, were collected. The Pediatric Quality of Life Inventory Measurement Models 4.0 (PedsQL™ 4.0) was utilized to assess the HRQOL of these children and to explore correlations among different dimensions of quality of life. General linear regression models were applied to analysis of influencing factors of the HRQOL of children with IgAV.

**Results:**

The health-related quality of life scores in all dimensions for children with IgAV were lower compared to those of healthy children. Recurrence of IgAV was negatively correlated with all dimension scores and the total HRQOL score (*P* < 0.05). Children with exercise-induced onset showed lower physiological functioning scores compared to those without exercise-induced onset. Emotional functioning scores were lower in children with self-funded medical care compared to those with Medicare. Children with medication duration longer than 1 week exhibited higher social functioning scores.

**Conclusions:**

The HRQOL of IgAV children is lower than that of healthy children. Relapse, exercise, the payment method and regularity of medications affect children's health-related quality of life.

## 1 Introduction

IgA vasculitis (IgAV) is a common systemic vascular inflammation with small vessel vasculitis as the main lesion in childhood, with an annual incidence of about 3–26.7 per 100,000 in children, and common in aged 5–7 years, males are more affected than that of females, and the disease is more likely in winter. The clinical manifestations are nonthrombocytopenic purpura, accompanied by joint swelling and pain or limited joint mobility, abdominal pain or hematochezia, hematuria, proteinuria, etc (1). It can cause systemic multi-organ dysfunction and is prone to recurrence and requires multiple and long-term treatment (1).

With the shift to the biopsychosocial model of medicine, there is growing recognition of the importance of psychological well-being and social functioning in disease management. Health-Related Quality of Life (HRQOL) has emerged as a crucial measure beyond physical health, serving as a robust indicator for assessing population health, evaluating resource allocation efficiency, and guiding clinical treatment decisions (2, 3). Silva et al. (4) conducted a meta-analysis of 21 studies comparing HRQOL in pediatric patients vs. healthy children, highlighting lower HRQOL in pediatric patients and emphasizing the need for targeted evaluation and intervention in pediatric HRQOL. However, domestic research on children's quality of life has primarily focused on specific diseases such as tumors, hematological disorders (both benign and malignant), cerebral palsy, autism, and obesity (5–10).

Some studies have explored the reduce of HRQOL in IgAV nephritis (IgAVN) and severe IgAV (11–13), research encompassing overall HRQOL and influencing factors in the broader population of children with IgAV remains limited.

IgAV can reduce the physiological function score by damaging the skin, joints, digestive tract, and kidneys.It may also have impact on the children's school performance by limit the school learning time and projects because of taking medication for a long time and repeated treatments in the later stage of the disease.Large-scale purpura or nephropathy requiring hormone therapy, which may affect the emotional and social functioning because of the changes in appearance and low self-esteem.Therefore, the health related quality of life of children with IgAV urgently needs to be concerned.

Against this backdrop, this study aims to investigate the current HRQOL in children diagnosed with IgAV and explore factors influencing it, aiming to provide insights for post-discharge rehabilitation of these children.

## 2 Materials and methods

### 2.1 Study subjects

Children diagnosed with IgAV at Bazhong Central Hospital from January 2019 to January 2023, constituting the observation group. The children without congenital malformations, cancer and other acute and chronic diseases undergoing health check-ups during the same period were included into control group. This study received approval from the hospital's medical ethics committee [Audit No.: Ba Shi Yi Lun (2024) No. 6], and informed consent was obtained from all participants' guardians.

#### 2.1.1 Inclusion critrria

Aged 2–14 years old; diagnose based on the 2019 European consensus-based recommendations for the diagnosis and treatment of IgAV (14); Recurrence defined as the reappearance of IgAV symptoms or signs at least 4 weeks after the initial resolution.

#### 2.1.2 Exclusion criteria

Congenital metabolic diseases; Other malformations, severe chronic diseases, or malignancies requiring frequent hospital visits;Cognitive impairments in children or parents affecting the understanding of questionnaire content; refusal to participate in the survey and loss to follow-up.

#### 2.1.3 Sample size estimates

R 4.2.3 software was used to estimate the final sample size of no less than 23–24 people in the control group and observation group respectively.

### 2.2 Research methods

HRQOL was assessed using the Pediatric Quality of Life Inventory Measurement Models 4.0 (PedsQL™ 4.0).This Model has high reliability and validity, and is suitable for horizontal comparative studies of healthy children and pediatric patients with acute and chronic diseases (10). It contains four dimensions: physiological function, emotional function, social function, and school performance. The scale is divided into questionnaires for children aged 5–7, 8–12 and 13–18 years and parent questionnaires for children aged 2–4, 5–7, 8–12 and 13–18 years.The parent questionnaire for children aged 2–4 years contains 21 items, and the rest of the questionnaires contain 23 items. The PedsQL directions instruct respondents to review a list of problem statements and rate the degree to which each has been experienced over the past month. Overall HRQOL and domain scores are the average score of the included items and range from 0 to 100. The higher the score, the better the quality of life (10).

The general data, clinical data, and PedsQL were published on the questionnaire star software, and the children in the observation group and their parents filled in the questionnaires 1 month after recovery and discharge. The children in the control group and their parents filled out the questionnaire at the child care clinic.

Sociodemographic and clinical data were collected to analyze:

1. Current quality of life among children with IgAV.
2. Correlations among different dimensions of quality of life in children with IgAV.
3. Factors influencing the quality of life in children with IgAV.

### 2.3 Statistical methods

Data were analyzed using SPSS 25. Quantitative data with a skewed distribution were described using the median and interquartile range [M (P25, P75)], while normally distributed quantitative data were described using the mean ± standard deviation ($\bar{X}\pm S$). Qualitative data were presented as counts and proportions (*n*, %). For normally distributed quantitative data, independent sample *T*-tests or *F*-tests were used to compare between two or more groups, while the Wilcoxon rank test was employed for skewed quantitative data comparisons. Categorical variables were analyzed using the chi-square test (*χ*^2^). Health-Related Quality of Life (HRQOL) scores in each dimension and the total score served as dependent variables. Linear correlation analysis was performed for normally distributed quantitative data, while rank correlation analysis was used for skewed quantitative data or categorical variables. Multivariate linear regression analysis was conducted for variables showing statistically significant differences in univariate analysis. A *P*-value of <0.05 was considered statistically significant.

## 3 Results

A total of 239 children with IgA vasculitis met the inclusion criteria during the study period, finally only 114 patients met inclusion criteria were included in the survey, 112 valid questionnaires for children and 114 questionnaires for parents; 51 healthy children undergoing health check-ups during the same period were enrolled in control group, 31 valid questionnaires for children and 51 questionnaires for parents.

In the observation group, there were 68 males (59.65%) and 46 females (40.35%), and 20 cases (17.54%) aged < 7 years old and 94 cases (82.46%) aged ≥ 7 years old. The clinical classification was simple type in 39 cases (34.21%), abdominal type in 17 cases (14.91%), articular type in 31 cases (27.19%), renal type in 11 cases (9.65%), and mixed type in 16 cases (14.04%). There were 36 cases relapsed (31.58%) and 78 cases unrelapsed (68.42%). In the control group, there were 30 males (58.8%) and 21 females (41.2%), 14 cases (27.5%) aged < 7 years old and 37 cases (72.5%) aged ≥ 7 years old.

### 3.1 Comparative analysis of quality of life between children with IgAV and healthy children

The results showed that both the self-scores and parental scores of the HRQOL of the children with IgAV in the observation group were lower than those in the control group. Since the PedsQL of children aged 2–4 years only had parent's questionnaire,the score of parent's was used to compare with the healthy controls.

After adjusting for age and gender, the HRQOL in all dimensions and the total score for children with IgAV were lower than those of healthy children with physiological functioning (87.47 vs. 96.64, *P* < 0.01), emotional functioning (83.83 vs. 96.19, *P* < 0.01), social functioning (89.10 vs. 97.94, *P* < 0.01), and school functioning (75.63 vs. 96.19, *P* < 0.01), total score (84.01 vs. 96.74, *P* < 0.01) (Table 1).

**Table 1:** Comparison of quality of life between observation and control groups.

Item	Control group (*n* = 51)	Observation group (*n* = 114)	Statistic	*P* Value
Age	7.86 ± 2.55	8.43 ± 1.09	1.39^a^	0.168
Gender				
Male	31 (60.8%)	68 (59.6%)	0.02^b^	0.891
Female	20 (39.2%)	45 (40.4%)		
Physical Functioning	96.64 ± 3.15	87.47 ± 14.30	6.50^a^	<0.01
Emotional Functioning	96.19 ± 2.76	83.83 ± 17.09	7.51^a^	<0.01
School Functioning	96.19 ± 3.27	75.63 ± 18.00	11.77^a^	<0.01
Social Functioning	97.94 ± 2.39	89.10 ± 13.50	6.76^a^	<0.01
Total Score	96.74 ± 1.45	84.01 ± 13.57	9.89^a^	<0.01

^a^ Indicates the *t*-test statistic.

^b^ Indicates the chi-square test statistic.

### 3.2 Correlation analysis of HRQOL dimensions in children with IgAV

There were positive correlations between the dimensions of physical functioning, emotional functioning, social functioning, and school functioning in the HRQOL of children with IgAV (*P* < 0.05) (Table 2).

**Table 2:** Correlation analysis of various dimensions of quality of life in children with igAV.

Dimension	Physical functioning	Emotional functioning	Social functioning	School functioning	Total score
Physical Functioning	1				
Emotional Functioning	0.733**	1			
Social Functioning	0.727**	0.787**	1		
School functioning	0.620**	0.582**	0.542**	1	
Total Score	0.881**	0.897**	0.868**	0.813**	1

Pearson correlation, **P < 0.01, significant correlation.

### 3.3 Univariate analysis of influencing factors on quality of life dimensions in children with IgAV

A univariate analysis was conducted for each quality of life dimension and the total score in children with IgAV. The results indicated that age, academic performance, medical insurance payment method, impact on family economy, repeated hospitalizations, cumulative hospitalization costs, recurrence, food-induced onset, exercise-induced onset, seasonal changes-induced onset, and persistent proteinuria were all correlated with physical functioning, emotional functioning, social functioning, school functioning, and the total score (*P* < 0.05). Additionally, joint swelling and pain affecting daily life, and the presence of melena at onset were correlated with physical functioning (*P* < 0.05); age at first onset and severity of abdominal pain were correlated with emotional functioning (*P* < 0.05); severity of abdominal pain and duration of medication were correlated with social functioning (*P* < 0.05); family monthly income, severity of abdominal pain, and duration of medication were correlated with school functioning (*P* < 0.05); and age at first onset, joint swelling and pain affecting daily life, severity of abdominal pain, presence of melena, and duration of medication were correlated with the total score (*P* < 0.05) (Table 3).

**Table 3:** Univariate analysis of influencing factors of health-related quality of life in children with igAV.

Item	Cases (*n*, %)	Physical functioning	Emotional functioning	Social functioning	School functioning	Total score
Age						
<7 years old	20 (17.54%)	3.627^a,^**	5.094^a,^**	5.562^a,^**	2.877^a,^**	5.893^a,^**
≥7 years old	94 (82.46%)					
Academic Performance						
Excellent	39 (34.21%)	2.708^b,^*	4.731^b,^*	4.514^b,^*	4.762^b,^**	6.252^b,^**
Good	48 (42.11%)					
Average	21 (18.42%)					
Poor	6 (5.26%)					
Medical Payment Method						
Self-paying	39 (34.21%)	2.220^a,^*	3.109^a,^**	2.982^a,^**	2.545^a,^*	3.066^a,^**
Medical Insurance	75 (65.79%)					
Family Monthly Income						
≤1,000 yuan	4 (3.51%)				3.042^b,^*	
1,000–3,000 yuan	23 (20.18%)					
3,000–5,000 yuan	38 (33.33%)					
≥5,000 yuan	49 (42.98%)					
Degree of Economic Impact						
None or minimal impact	56 (49.12%)	2.536^a,^*	3.339^a,^**	3.076^a,^**	3.907^a,^**	3.840^a,^**
Significant impact	58 (50.88%)					
Repeated Hospitalization						
No	76 (66.67%)	3.679^a,^**	3.063^a,^**	3.465^a,^**	2.913^a,^**	3.634^a,^**
Yes	38 (33.33%)					
Cumulative Hospital Costs						
≤1,000 yuan	27 (23.68%)	4.219^b,^**	5.190^b,^**	2.648^b,^*	2.932^b,^*	4.635^b,^**
1,000–3,000 yuan	41 (35.96%)					
3,000–5,000 yuan	12 (10.53%)					
5,000–10,000 yuan	13 (11.4%)					
≥10,000 yuan	21 (18.42%)					
Recurrence						
No	78 (68.42%)	5.103^a,^**	5.435^a,^**	6.403^a,^**	4.871^a,^**	6.147^a,^**
Yes	36 (31.58%)					
Age at first onset						
2–3 years old	20 (17.54%)		2.645^b,^*			4.095^b,^*
4–7 years old	47 (41.23%)					
8–14 years old	47 (41.23%)					
Food-induced onset						
No	67 (58.78%)	3.529^a,^**	2.822^a,^**	4.111^a,^**	2.622^a,^*	3.670^a,^**
Yes	47 (41.22%)					
Seasonal Changes-induced onset						
No	88 (77.19)	2.468^a,^*	2.584^a,^*	2.640^a,^*	2.140^a,^*	2.444^a,^*
Yes	26 (22.81%)					
Exercise-induced onset						
No	82 (71.93%)	3.660^a,^**	2.902^a,^**	2.956^a,^**	3.183^a,^**	3.479^a,^**
Yes	32 (28.07%)					
Joint Swelling and pain affecting daily life						
No	37 (32.46%)	2.376^a,^*				2.286^a,^*
Yes	77 (67.54%)					
Severity of abdominal pain						
None or mild	77 (67.54%)		1.979^a,^*	2.304^c,^*	2.513^a,^*	2.310^a,^*
Severe or persistent	37 (32.46%)					
Presence of Melena at onset						
No	77 (67.54%)	2.268^a,^*				2.099^a,^*
Yes	37 (32.46%)					
Persistent Proteinuria						
No	88 (77.19%)	2.145^a,^*	2.721^a,^**	2.722^a,^*	2.872^a,^**	2.642^a,^*
Yes	26 (22.81%)					
Medication Duration						
≤7 days	12 (10.53%)			2.199^c,^*	2.044^a,^*	2.576^c,^**
>7 days	102 (89.47%)					

* Indicates *P* < 0.05.

** Indicates *P* < 0.01.

^a^ Indicates *t*-test statistic.

^b^ Indicates *F*-test statistic.

^c^ Indicates rank-sum test statistic.

### 3.4 Multivariate analysis of influencing factors on quality of life dimensions in children with IgAV

**Figure 1:**
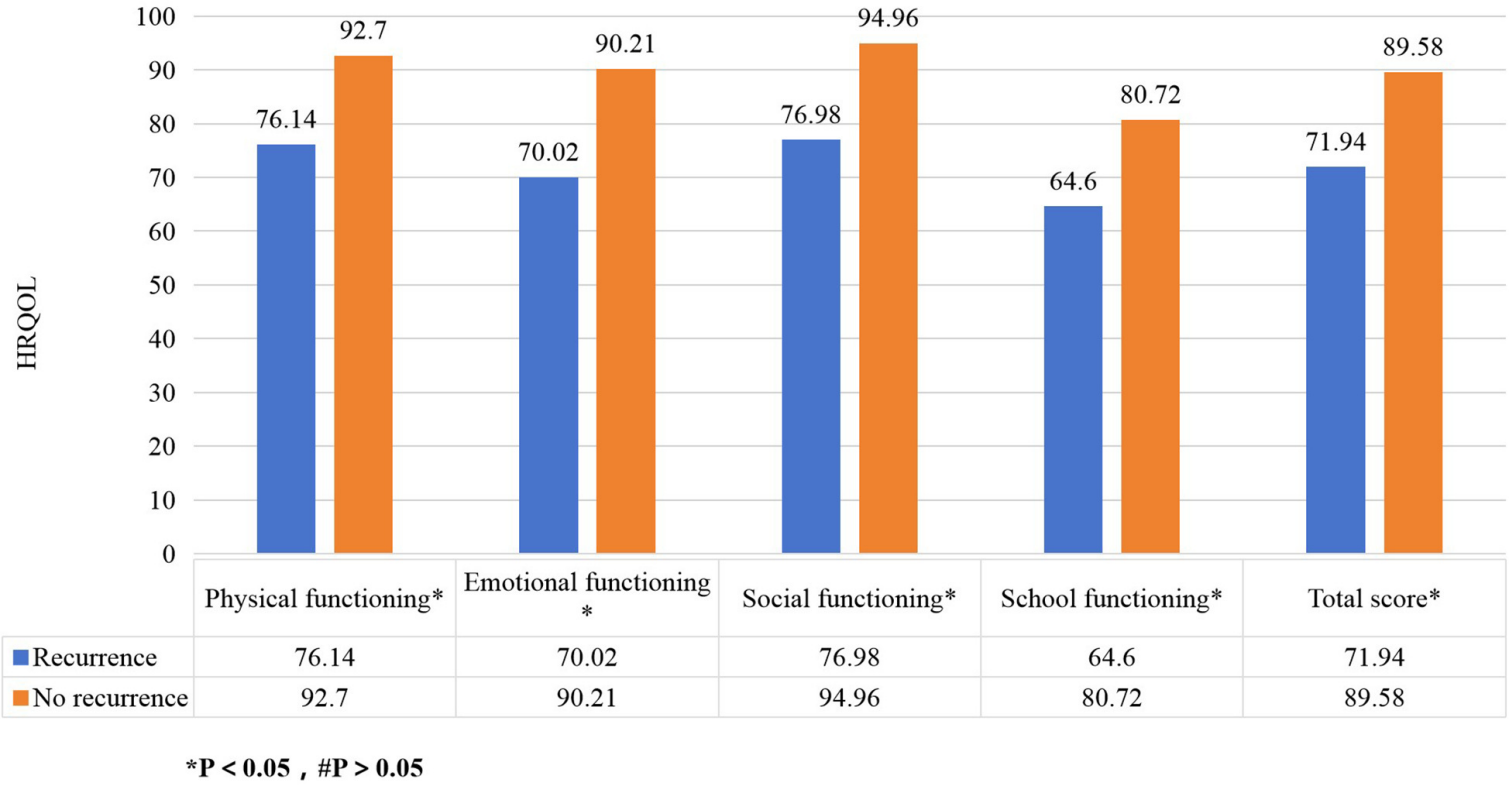
The impact of recurrence on HRQOL in children with IgAV. The figure shows recurrence of IgAV reduced HRQOL scores across all dimensions.

**Figure 2:**
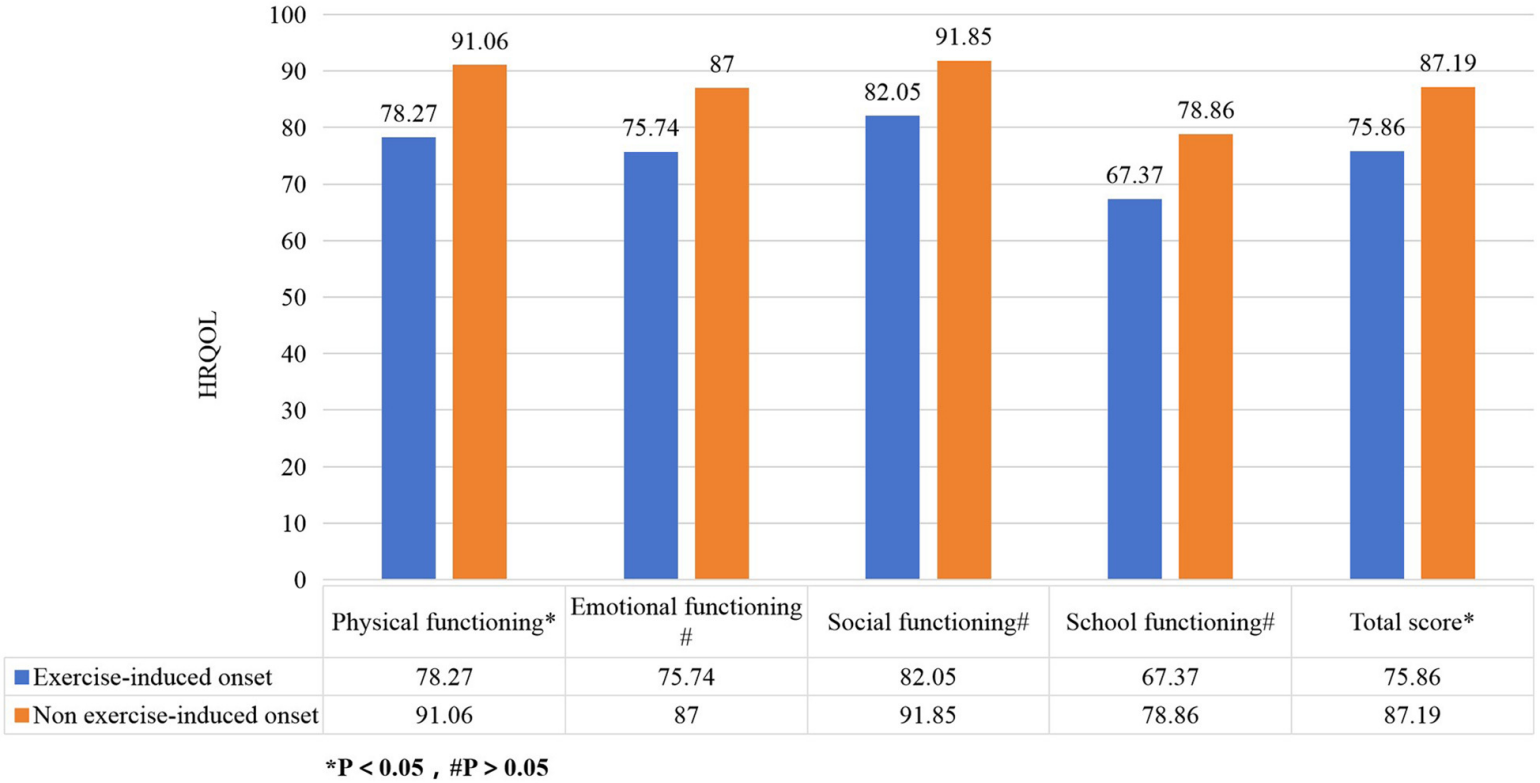
Effect of exercise on HRQOL in children with IgA vasculitis. The figure shows that children with exercise-induced onset had lower physical functioning scores compared to those without exercise limitations.

**Figure 3:**
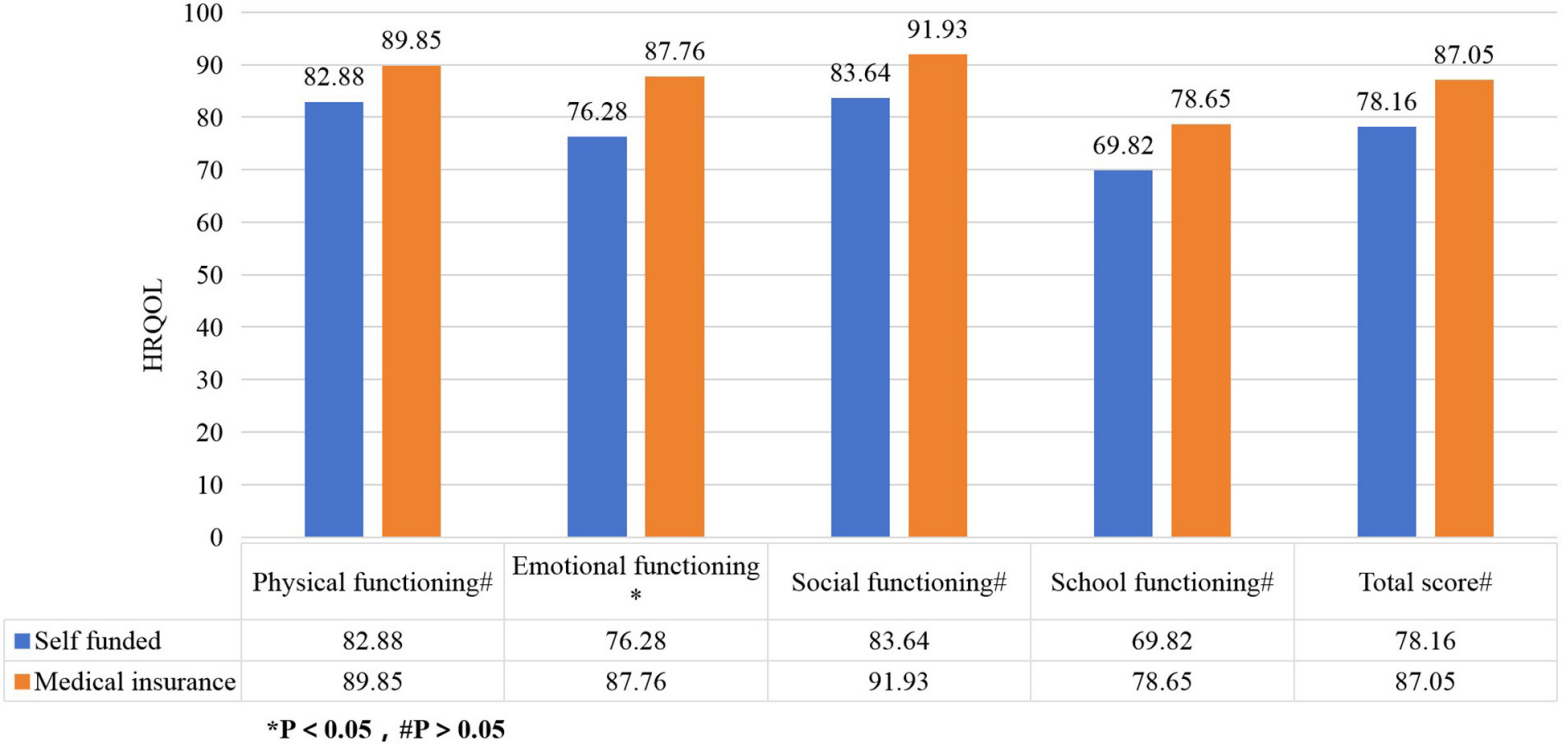
Effect of medical payment methods on HRQOL in children with IgAV. The figure shows that children covered by medical insurance exhibited higher emotional functioning scores compared to self-paying patients.

**Figure 4:**
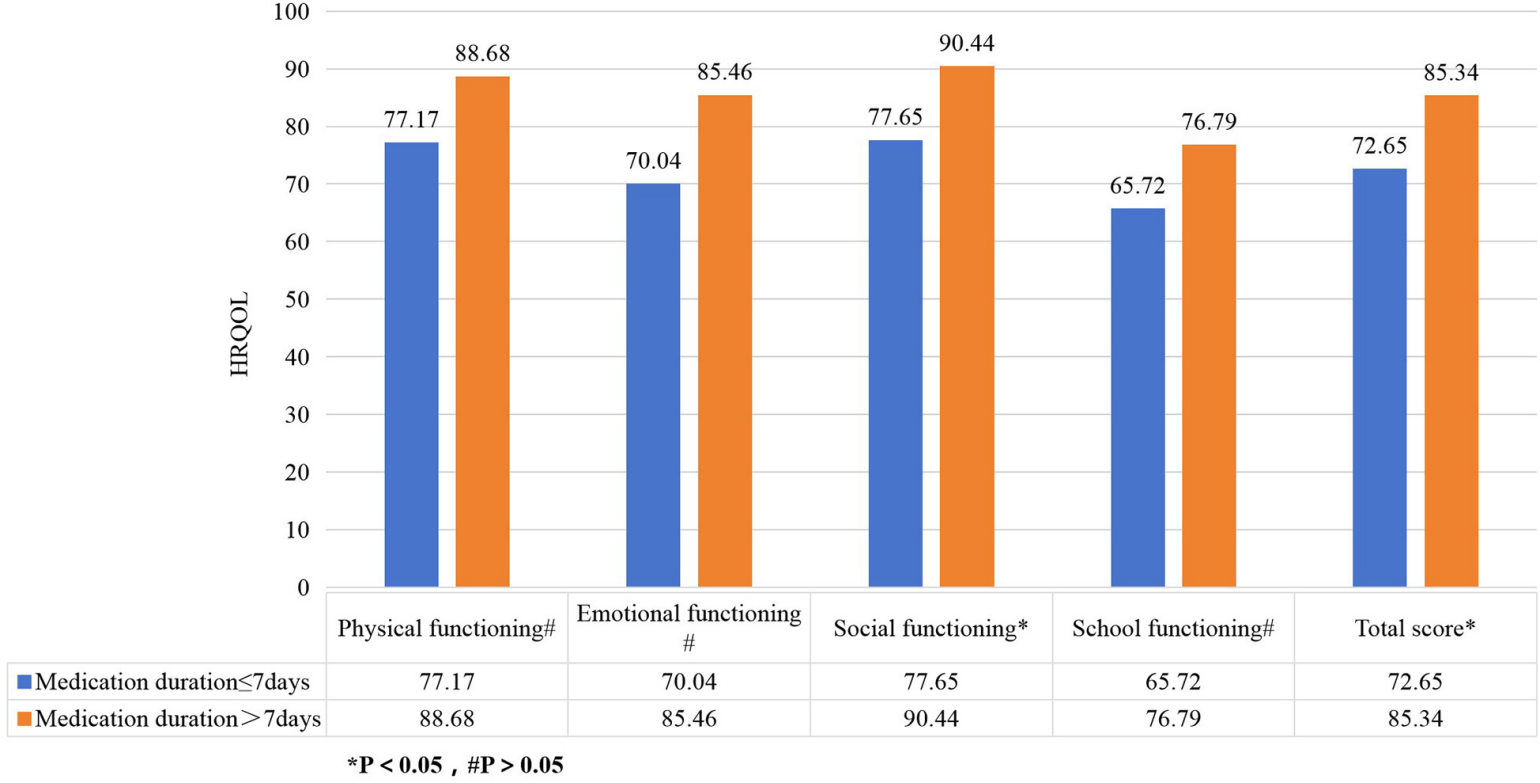
Effect of medication time on HRQOL in children with IgAV. The figure shows that children who adhered to medication for more than one week showed higher social functioning and total scores compared to those with shorter adherence durations.

The recurrence of IgAV significantly reduced HRQOL scores across all dimensions with physiological functioning (76.14 vs. 92.70, *P* < 0.05), emotional functioning (70.02 vs. 90.21, *P* < 0.05), social functioning (76.98 vs. 94.96, *P* < 0.05), and school functioning (64.60 vs. 80.72, *P* < 0.05),total score (71.94 vs. 89.58, *P* < 0.05) (Figure 1). Children with exercise-induced onset had lower physical functioning scores compared to those without exercise limitations (78.27 vs. 91.05, *P* < 0.05) (Figure 2). Children covered by medical insurance exhibited higher emotional functioning scores compared to self-paying patients (87.76 vs. 76.28, *P* < 0.05) (Figure 3); Children who adhered to medication for more than one week showed higher social functioning (90.44 vs. 77.65, *P* < 0.05) and total scores (85.34 vs. 72.65, *P* < 0.05) compared to those with shorter adherence durations (Figure 4).

## 4 Discussion

This study found that children with IgAV had lower HRQOL scores in physical functioning, emotional functioning, social functioning, and school functioning, as well as the total score, compared to healthy children. This indicates that IgAV significantly impacted the quality of life in children. These findings are consistent with Mańczak et al. (15–18), who reported that chronic diseases negatively affected children's quality of life, underscoring the importance of addressing HRQOL in children with IgAV alongside disease management.

The recurrence of IgAV had an impact on each dimension and the total score of HRQOL. IgAV is a self-limiting disease, but 2.5–66.2% of children may experience recurrence within 3–6 months after the initial onset (19). Recurrence may lead to chronic kidney damage and reduced physical functioning, and it can also cause anxiety and depression, thereby lowering emotional functioning scores. Recurrence-associated physical dysfunction and pain decrease happiness and social desires, while frequent medical visits due to recurrence can disrupt learning and school functioning. These findings are consistent with those of Lindman et al. (20). Therefore, recurrence is significantly negatively correlated with HRQOL in children with IgAV, underscoring the importance of monitoring changes in quality of life in clinical practice and making efforts to prevent recurrence.

Children whose onset was induced by exercise had lower physical functioning scores compared to those without exercise restrictions. Increased vascular fragility in children with IgAV means that frequent vigorous exercise can elevate the risk of small vessel damage between muscles, resulting in rashes, joint or muscle bleeding, swelling, and pain, which in turn restricts activity and diminishes physical functioning (21). These results are consistent with findings by Grasaas et al. (22, 23) regarding the impact of pain on children's quality of life. However, there are also reports suggesting that appropriate exercise can improve the quality of life in patients with chronic diseases (5, 24). This study also found that non-exercise-induced morbidity did not significantly affect the HRQOL of children with IgAV. Therefore, excessive restrictions on exercise should be avoided in these patients to prevent negative impacts on physical functioning.

The influence of payment methods on children's emotional functioning may be overlooked in clinical practice. Children without medical insurance support may pay less attention to their illness, exhibit lower treatment adherence, and experience feelings of inferiority and anxiety, which can impact their emotional functioning scores (25). This underscores the importance of expanding medical insurance coverage in future medical practice.

Children who adhered to long-term medication had higher social functioning scores compared to those with poor adherence. Adequate medication duration gradually restores their immune function, reducing the likelihood of recurrence. The sense of accomplishment from recovery enhances self-esteem and social engagement, thereby improving their quality of life (26). Children with poor adherence may discontinue medication once acute symptoms subside, leaving immune abnormalities unresolved and increasing the risk of recurrence, along with feelings of anxiety, inferiority, distrust, and social withdrawal (27). This highlights the importance of emphasizing medication adherence during treatment and follow-up. However, this study only conducted a questionnaire survey on children who had been ill for one month, and further research is needed to determine if long-term medication affects the HRQOL of children with IgAV who require months or even longer on medication.

Other studies on chronic diseases in children have indicated that quality of life is influenced by the social characteristics of the parents (28, 29). However, these factors were not found to be significant in this study. This may be attributed to the fact that 76% of the primary caregivers in this study were mothers, who generally have higher education levels due to societal advancements.

## 5 Conclusion

The HRQOL of children with IgAV was lower than that of healthy children. There was a positive correlation between the dimensions of quality of life and with the total score. IgAV recurrence has an impact on all dimensions of quality of life and overall quality of life. Exercise is the cause of IgAV and affects the physiological function of children. The payment method affects the emotional function of children, and the social function of children is affected by adherence to medication.Efforts should focus on reducing recurrence rates, providing appropriate guidance on physical activity, expanding medical insurance coverage, and promoting medication adherence to improve the HRQOL of children with IgAV.

### 5.1 Deficiencies and prospects

This study is limited by its single-center design, use of a single quality of life research scale, and a small sample size. Future research should aim for multi-center studies using multiple quality of life scales.

## Data Availability

The original contributions presented in the study are included in the article/Supplementary Material, further inquiries can be directed to the corresponding author.

## References

[1] Fretzayas A Sionti I Moustaki M Papadimitriou A Nicolaidou P. Henoch-Schönlein purpura: a long-term prospective study in Greek children. J Clin Rheumatol. (2008) 14:324–31. 10.1097/RHU.0b013e31817a240a 18703982

[2] Chonghua W. Discussion on some important issues in quality of life research (part 1). Chin J Behav Med Sci. (1999) 1:66. 10.3760/cma.j.issn.1674-6554.1999.01.041

[3] Fang Jiqian WC Yuantao H. Overview of research on health-related quality of life. Chin J Rehabil Med. (2000) 1:39–43. 10.3969/j.issn.1001-1242.2000.01.014

[4] Silva N Pereira M Otto C Ravens-Sieberer U Canavarro MC Bullinger M. Do 8- to 18-year-old children/adolescents with chronic physical health conditions have worse health-related quality of life than their healthy peers? A meta-analysis of studies using the KIDSCREEN questionnaires. Qual Life Res. (2019) 28:1725–50. 10.1007/s11136-019-02189-7 31055778

[5] Zhao Y Chen YP Wu YQ Bao BY Fan H. Effect of physical activity on depression symptoms in patients with IgA nephropathy. J Int Med Res. (2020) 48:300060519898008. 10.1177/0300060519898008 31948307 PMC7113810

[6] Zhi L Lu Z Juan B Jing F. Analysis of quality of life and influencing factors in children with severe β—thalassemia in Guangxi. Int Med. (2023) 18:377–81. 10.16121/j.cnki.cn45-1347/r.2023.04.16

[7] Ying C Jizhao G Wenpeng W. Comparison of quality of life and analysis of influencing factors in children with benign and malignant hematological diseases after hematopoietic stem cell transplantation. Chin J Pediatr Hematol Oncol. (2023) 28:248–52. 10.3969/j.issn.1673-5323.2023.04.007

[8] Xiaolan Y. The effect of acupoint implantation of protein thread therapy on motor function in children with cerebral palsy. Chin Med Mod Dis Edu of China. (2021) 19:103–5. 10.3969/j.issn.1672-2779.2021.15.038

[9] Xinping L Zhuo C Hongxiang M Zanping P. Observation on the therapeutic effect of probiotics in autism and their impact on oxidative stress status. China Health Stand Manag. (2022) 13:139–43. 10.3969/j.issn.1674-9316.2022.17.030

[10] Yiyun L Yuantao H. Overview of the child quality of life measurement scale system. Chin J Behav Med Neurosci. (2005) 14:1128–9. 10.3760/cma.j.issn.1674-6554.2005.12.037

[11] Mizerska-Wasiak M Adamczuk D Cichoń-Kawa K Miklaszewska M Szymanik-Grzelak H Pietrzyk JA Health-related quality of life in children with immunoglobulin A nephropathy—results of a multicentre national study. Arch Med Sci. (2021) 17:84–91. 10.5114/aoms.2020.100367 33488859 PMC7811315

[12] Murphy SL Mahan JD Troost JP Srivastava T Kogon AJ Cai Y Longitudinal changes in health-related quality of life in primary glomerular disease: results from the CureGN study. Kidney Int Rep. (2020) 5:1679–89. 10.1016/j.ekir.2020.06.041 33102960 PMC7569685

[13] Lei Z. Observation of the therapeutic effect of methylprednisolone combined with immunoglobulin shock therapy on severe children with allergic purpura. Elec J Clin Med Lit. (2019) 6:75–77. 10.16281/j.cnki.jocml.2019.45.062

[14] Ozen S Marks SD Brogan P Groot N de Graeff N Avcin T European consensus-based recommendations for diagnosis and treatment of immunoglobulin A vasculitis-the SHARE initiative. Rheumatology. (2019) 58:1607–16. 10.1093/rheumatology/kez041 30879080

[15] Mańczak M Rutkowska-Sak L Raciborski F. Health-related quality of life in children with juvenile idiopathic arthritis—child’s and parent’s point of view. Reumatologia. (2016) 54:243–50. 10.5114/reum.2016.63665 27994269 PMC5149572

[16] Helseth S Haraldstad K Christophersen KA. A cross-sectional study of health related quality of life and body mass index in a Norwegian school sample (8–18 years): a comparison of child and parent perspectives. Health Qual Life Outcomes. (2015) 13:47. 10.1186/s12955-015-0239-z 25884676 PMC4396077

[17] Gerson AC Wentz A Abraham AG Mendley SR Hooper SR Butler RW Health-related quality of life of children with mild to moderate chronic kidney disease. Pediatrics. (2010) 125:e349–357. 10.1542/peds.2009-0085 20083528 PMC3663134

[18] Kiliś-Pstrusińska K Medyńska A Chmielewska IB Grenda R Kluska-Jóźwiak A Leszczyńska B Perception of health-related quality of life in children with chronic kidney disease by the patients and their caregivers: multicentre national study results. Qual Life Res. (2013) 22:2889–97. 10.1007/s11136-013-0416-7 23595412 PMC3853413

[19] Kaili Z Yanping H. Research progress on risk factors for recurrent allergic purpura in children. Res Maternal Child Health China. (2021) 32:1882–5. 10.3969/j.issn.1673-5293.2021.12.030

[20] Lindman JP Lewis LS Accortt N Wiatrak BJ. Use of the pediatric quality of life inventory to assess the health-related quality of life in children with recurrent respiratory papillomatosis. Ann Otol Rhinol Laryngol. (2005) 114:499–503. 10.1177/000348940511400701 16134343

[21] Yunhe Y Bing W. The clinical significance of reducing exercise intensity in the treatment of allergic purpura. Jilin Med. (2015) 36:1420–1. 10.3969/j.issn.1004-0412.2015.07.097

[22] Grasaas E Helseth S Fegran L Stinson J Småstuen M Haraldstad K. Health-related quality of life in adolescents with persistent pain and the mediating role of self-efficacy: a cross-sectional study. Health Qual Life Outcomes. (2020) 18:19. 10.1186/s12955-020-1273-z 32000787 PMC6993393

[23] Haraldstad K Christophersen KA Helseth S. Health-related quality of life and pain in children and adolescents: a school survey. BMC Pediatr. (2017) 17:174. 10.1186/s12887-017-0927-4 28738818 PMC5525195

[24] Nicolodi GV Della Méa Plentz R Righi NC Stein C. Effects of aerobic exercise on patients with pre-dialysis chronic kidney disease: a systematic review of randomized controlled trials. Disabil Rehabil. (2022) 44:4179–88. 10.1080/09638288.2021.1900929 34033723

[25] Hossain MJ Xie S. Patient features and survival of pediatric aplastic anemia in the USA: a large institution experience. J Public Health. (2019) 41:329–37. 10.1093/pubmed/fdy104 PMC6662081 29901745

[26] Liao C Chen J. Study on the correlation between quality of life and self-awareness of school-age children with chronic diseases. J Nurs. (2011) 26:38–40. 10.3870/hlxzz.2011.11.038

[27] Yanan W Zhijun W Lijian C Fangfang Y Lihua L Xuemei H Research progress on the impact of shame on social avoidance and distress in epilepsy patients. Epilepsy J. (2024) 10:32–8. 10.7507/2096-0247.202311013

[28] Changjun W. Applicability of PedsQL™ 4.0 in the study of quality of life in children with functional constipation and their families: The fourth military medical university. (2015).

[29] von Rueden U Gosch A Rajmil L Bisegger C Ravens-Sieberer U. Socioeconomic determinants of health related quality of life in childhood and adolescence: results from a European study. J Epidemiol Community Health. (2006) 60:130–5. 10.1136/jech.2005.039792 16415261 PMC2566139

